# 6-(4-Meth­oxy­phen­yl)-6a-nitro-6,6a,6b,7,8,9,10,12a-octa­hydro­spiro­[chromeno[3,4-*a*]indolizine-12,3′-indolin]-2′-one

**DOI:** 10.1107/S1600536813015341

**Published:** 2013-06-08

**Authors:** Seenivasan Karthiga Devi, Thothadri Srinivasan, Jonnalagadda Naga Siva Rao, Raghavachary Raghunathan, Devadasan Velmurugan

**Affiliations:** a Centre of Advanced Study in Crystallography and Biophysics, University of Madras, Guindy Campus, Chennai 600 025, India; b Department of Organic Chemistry, University of Madras, Guindy Campus, Chennai 600 025, India

## Abstract

In the title compound, C_29_H_27_N_3_O_5_, the hydropyran ring adopts an envelope conformation with the methine C atom bearing the *para*-meth­oxy­benzene ring as the flap. The central pyrrolidine ring has a twist conformation on the N—C bond involving the spiro C atom. The piperidine ring adopts a chair conformation. An intra­molecular C—H⋯O contact closes an *S*(7) ring. In the crystal, inversion dimers linked by C—H⋯O inter­actions generate *R*_2_^2^(18) loops and N—H⋯O hydrogen bonds connect the dimers into [100] chains.

## Related literature

For a related structure and background to 4*H*-chromene derivatives, see: Devi *et al.* (2013 ▶).
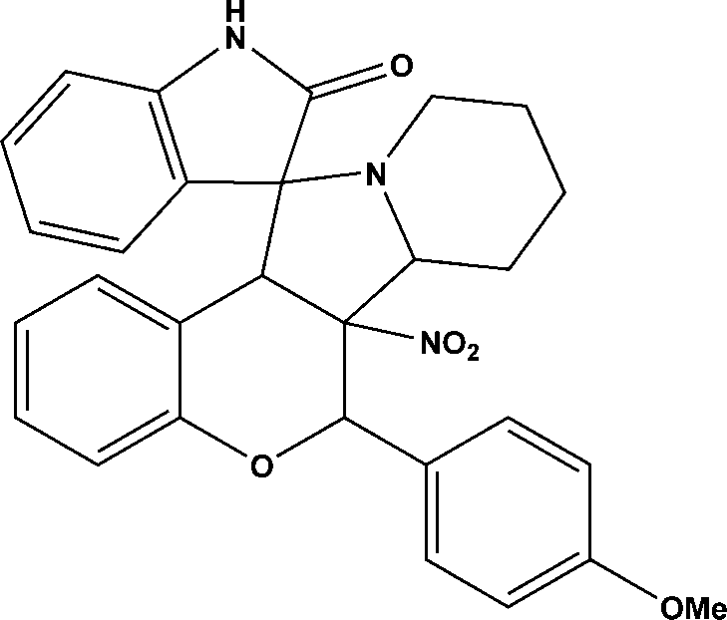


## Experimental

### 

#### Crystal data

C_29_H_27_N_3_O_5_
*M* *_r_* = 497.54
Triclinic, 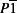

*a* = 9.3438 (3) Å
*b* = 11.3626 (4) Å
*c* = 13.5713 (4) Å
α = 68.687 (1)°
β = 88.284 (1)°
γ = 66.757 (1)°
*V* = 1222.49 (7) Å^3^
*Z* = 2
Mo *K*α radiation
μ = 0.09 mm^−1^
*T* = 293 K
0.30 × 0.25 × 0.20 mm

#### Data collection

Bruker SMART APEXII diffractometer
Absorption correction: multi-scan (*SADABS*; Bruker, 2008 ▶) *T*_min_ = 0.973, *T*_max_ = 0.982
17765 measured reflections
4964 independent reflections
4116 reflections with *I* > 2σ(*I*)
*R*_int_ = 0.027

#### Refinement

*R*[*F* ^2^ > 2σ(*F* ^2^)] = 0.042
*wR*(*F* ^2^) = 0.119
*S* = 1.03
4964 reflections
335 parameters
H-atom parameters constrained
Δρ_max_ = 0.22 e Å^−3^
Δρ_min_ = −0.23 e Å^−3^

### 

Data collection: *APEX2* (Bruker, 2008 ▶); cell refinement: *SAINT* (Bruker, 2008 ▶); data reduction: *SAINT*; program(s) used to solve structure: *SHELXS97* (Sheldrick, 2008 ▶); program(s) used to refine structure: *SHELXL97* (Sheldrick, 2008 ▶); molecular graphics: *ORTEP-3 for Windows* (Farrugia, 2012 ▶); software used to prepare material for publication: *SHELXL97* and *PLATON* (Spek, 2009 ▶).

## Supplementary Material

Crystal structure: contains datablock(s) global, I. DOI: 10.1107/S1600536813015341/hb7087sup1.cif

Structure factors: contains datablock(s) I. DOI: 10.1107/S1600536813015341/hb7087Isup2.hkl

Supplementary material file. DOI: 10.1107/S1600536813015341/hb7087Isup3.cml

Additional supplementary materials:  crystallographic information; 3D view; checkCIF report

## Figures and Tables

**Table 1:** Hydrogen-bond geometry (Å, °)

*D*—H⋯*A*	*D*—H	H⋯*A*	*D*⋯*A*	*D*—H⋯*A*
N3—H3*A*⋯O3^i^	0.86	2.24	3.085 (2)	167
C14—H14⋯O5^ii^	0.93	2.54	3.358 (2)	147
C8—H8⋯O5	0.98	2.40	3.234 (2)	143

Symmetry codes: (i) 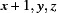; (ii) 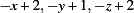.

**Fig. 1.**
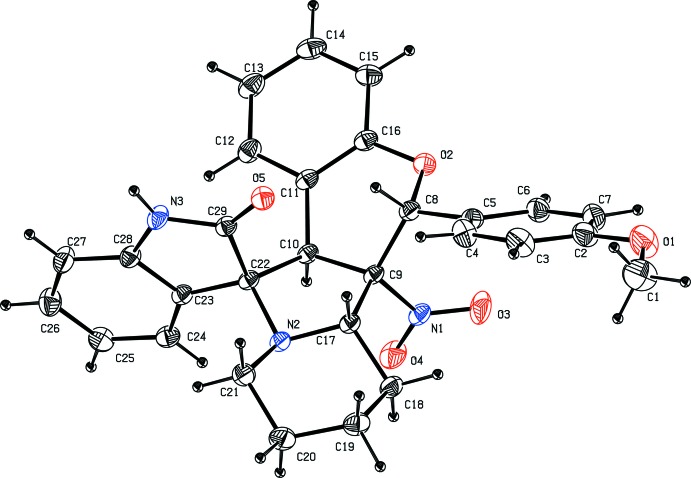
The molecular structure of the title compound, showing displacement ellipsoids drawn at the 30% probability level.

**Fig. 2.**
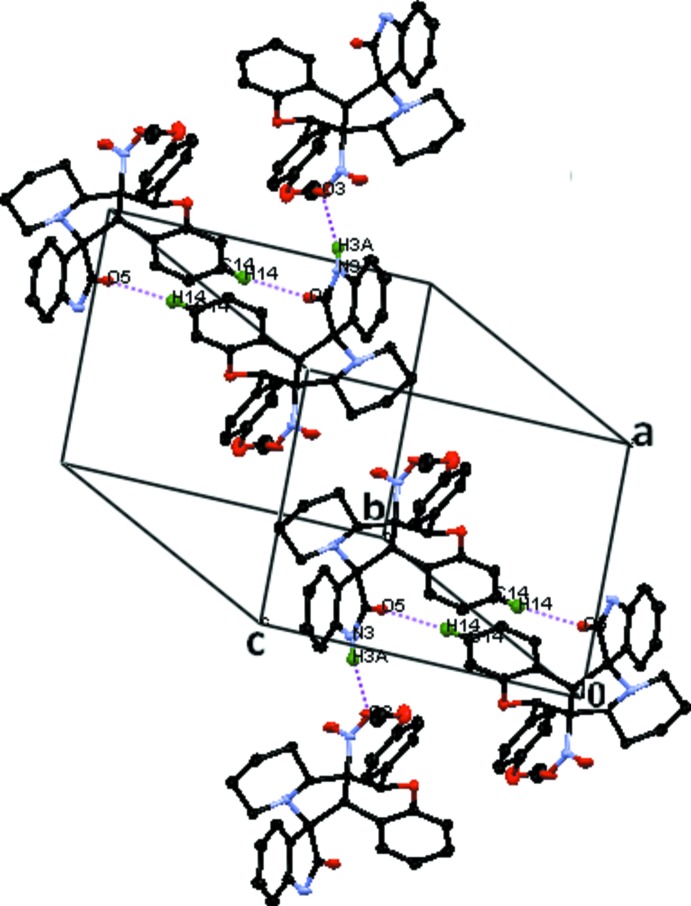
The crystal packing of the title compound viewed down b axis. H-atoms not involved in H-bonds have been excluded for clarity.

## supplementary crystallographic information

### Comment

As part of our ongoing studies of 4H-chromene derivatives (Devi *et al.*, 2013), we have synthesized the title compound (Fig. 1) and report herein its crystal structure.

The hydropyrrolidine ring adopts an *envelope* conformation, the piperidine ring adopts a *chair* conformation and the pyran ring adopts an *envelope* conformation. The pyrrolidine ring (N2/C9/C10/C17/C22) makes a dihedral angle of 86.78 (8)° with the other pyrrolidine ring (N3/C22/C23/C28/C29) which shows that they are almost orthogonal to each other. The pyrrolidine ring makes a dihedral angle of 29.65 (8)° with the pyran ring (O2/C8-C11/C16), it makes a dihedral angle of 8.88 (9)° with the piperidine ring (N2/C17-C21).

The other pyrrolidine ring (N3/C22/C23/C28/C29) makes a dihedral angle of 71.63 (8)° with the pyran ring, it makes a dihedral angle of 86.84 (8)° with the piperidine ring which shows that they are almost at right angles to each other. The dihedral angle between the pyran ring and the piperidine ring is 30.34 (8)° . The oxygen atom O5 attached with pyrrolidine ring deviates by -0.0200 (1)Å. The nitrogroup attached with the pyrrolidine ring makes a diherdal angle of 88.83 (1)° which shows it is in orthogonal orientation . The crystal packing features N—H···O, C—H···O hydrogen bonds and intramolecular C—H···O hydrogen bonds.

### Experimental

To a solution of isatin (1equiv) and piperidine-2-carboxylic acid (1.4 equiv) in dry toluene, was added 2-(4-methoxyphenyl)-3-nitro-2H-chromene (1equiv) under nitrogen atmosphere. The reaction mixture was refluxed for 24h in Dean-Stark apparatus to give the cycloadducts. After completion of the reaction as indicated by TLC, the solvent was evaporated under reduced pressure. The crude product was extracted with dichloromethane. The organic layer was dried with anhydrous sodium sulphate and concentrated in vacuo. Then the crude product was purified by column chromatography using hexane/EtOAc (7:3) as eluent. Colourless blocks were obtained by slow evaporation of a solution of the title compound in ethyl acetate at room temperature.

### Refinement

The hydrogen atoms were placed in calculated positions and treated as riding atoms: C—H = 0.93 Å to 0.97 Å, and N—H =0.86 Å with Uiso(H) = 1.5Ueq(C) for methyl H atoms and = 1.2Ueq(C) for other H atoms.

### Crystal data

**Table:**

C_29_H_27_N_3_O_5_	*Z* = 2
*M**_r_* = 497.54	*F*(000) = 524
Triclinic, *P*1	*D*_x_ = 1.352 Mg m^−^^3^
Hall symbol: -P 1	Mo *K*α radiation, λ = 0.71073 Å
*a* = 9.3438 (3) Å	Cell parameters from 4964 reflections
*b* = 11.3626 (4) Å	θ = 1.6–26.4°
*c* = 13.5713 (4) Å	µ = 0.09 mm^−^^1^
α = 68.687 (1)°	*T* = 293 K
β = 88.284 (1)°	Block, colourless
γ = 66.757 (1)°	0.30 × 0.25 × 0.20 mm
*V* = 1222.49 (7) Å^3^	

### Data collection

**Table:**

Bruker SMART APEXII diffractometer	4964 independent reflections
Radiation source: fine-focus sealed tube	4116 reflections with *I* > 2σ(*I*)
Graphite monochromator	*R*_int_ = 0.027
ω and φ scans	θ_max_ = 26.4°, θ_min_ = 1.6°
Absorption correction: multi-scan (*SADABS*; Bruker, 2008)	*h* = −11→11
*T*_min_ = 0.973, *T*_max_ = 0.982	*k* = −14→14
17765 measured reflections	*l* = −16→16

### Refinement

**Table:**

Refinement on *F*^2^	Primary atom site location: structure-invariant direct methods
Least-squares matrix: full	Secondary atom site location: difference Fourier map
*R*[*F*^2^ > 2σ(*F*^2^)] = 0.042	Hydrogen site location: inferred from neighbouring sites
*wR*(*F*^2^) = 0.119	H-atom parameters constrained
*S* = 1.03	*w* = 1/[σ^2^(*F*_o_^2^) + (0.0598*P*)^2^ + 0.279*P*] where *P* = (*F*_o_^2^ + 2*F*_c_^2^)/3
4964 reflections	(Δ/σ)_max_ < 0.001
335 parameters	Δρ_max_ = 0.22 e Å^−^^3^
0 restraints	Δρ_min_ = −0.23 e Å^−^^3^

### Special details

**Table:**

Geometry. All esds (except the esd in the dihedral angle between two l.s. planes) are estimated using the full covariance matrix. The cell esds are taken into account individually in the estimation of esds in distances, angles and torsion angles; correlations between esds in cell parameters are only used when they are defined by crystal symmetry. An approximate (isotropic) treatment of cell esds is used for estimating esds involving l.s. planes.
Refinement. Refinement of F^2^ against ALL reflections. The weighted R-factor wR and goodness of fit S are based on F^2^, conventional R-factors R are based on F, with F set to zero for negative F^2^. The threshold expression of F^2^ > 2sigma(F^2^) is used only for calculating R-factors(gt) etc. and is not relevant to the choice of reflections for refinement. R-factors based on F^2^ are statistically about twice as large as those based on F, and R- factors based on ALL data will be even larger.

### Fractional atomic coordinates and isotropic or equivalent isotropic displacement parameters (Å^2^)

**Table:**

	*x*	*y*	*z*	*U*_iso_*/*U*_eq_	
C1	0.2341 (3)	1.0337 (2)	0.38130 (16)	0.0993 (8)	
H1A	0.3106	1.0725	0.3690	0.149*	
H1B	0.1336	1.1041	0.3434	0.149*	
H1C	0.2654	0.9592	0.3564	0.149*	
C2	0.3493 (2)	0.87030 (17)	0.55807 (14)	0.0637 (5)	
C3	0.4983 (2)	0.82230 (18)	0.53115 (14)	0.0682 (5)	
H3	0.5192	0.8662	0.4634	0.082*	
C4	0.6175 (2)	0.70767 (16)	0.60584 (13)	0.0592 (4)	
H4	0.7191	0.6778	0.5883	0.071*	
C5	0.58892 (17)	0.63663 (15)	0.70583 (11)	0.0455 (3)	
C6	0.43845 (19)	0.68872 (17)	0.73146 (13)	0.0545 (4)	
H6	0.4165	0.6446	0.7988	0.065*	
C7	0.3206 (2)	0.80484 (19)	0.65897 (14)	0.0650 (5)	
H7	0.2207	0.8393	0.6783	0.078*	
C8	0.72252 (16)	0.51114 (14)	0.78326 (10)	0.0408 (3)	
H8	0.8151	0.5325	0.7724	0.049*	
C9	0.77152 (15)	0.36977 (13)	0.77426 (10)	0.0367 (3)	
C10	0.91910 (15)	0.26057 (13)	0.85468 (10)	0.0366 (3)	
H10	0.9014	0.1758	0.8865	0.044*	
C11	0.94489 (16)	0.29989 (14)	0.94587 (10)	0.0397 (3)	
C12	1.07826 (18)	0.21855 (17)	1.02158 (11)	0.0507 (4)	
H12	1.1586	0.1446	1.0117	0.061*	
C13	1.0931 (2)	0.2457 (2)	1.11079 (12)	0.0600 (4)	
H13	1.1843	0.1920	1.1595	0.072*	
C14	0.9731 (2)	0.35255 (19)	1.12802 (12)	0.0594 (4)	
H14	0.9817	0.3685	1.1896	0.071*	
C15	0.8405 (2)	0.43555 (17)	1.05410 (12)	0.0536 (4)	
H15	0.7598	0.5081	1.0652	0.064*	
C16	0.82846 (17)	0.40991 (15)	0.96263 (11)	0.0434 (3)	
C17	0.81967 (15)	0.37337 (14)	0.66400 (10)	0.0383 (3)	
H17	0.8471	0.4528	0.6321	0.046*	
C18	0.70480 (18)	0.38308 (17)	0.58150 (11)	0.0499 (4)	
H18A	0.6086	0.4661	0.5662	0.060*	
H18B	0.6790	0.3032	0.6082	0.060*	
C19	0.7827 (2)	0.38777 (19)	0.48083 (12)	0.0598 (4)	
H19A	0.7147	0.3871	0.4286	0.072*	
H19B	0.7975	0.4731	0.4505	0.072*	
C20	0.9400 (2)	0.26524 (19)	0.50478 (13)	0.0596 (4)	
H20A	0.9914	0.2758	0.4409	0.071*	
H20B	0.9232	0.1811	0.5244	0.071*	
C21	1.04685 (18)	0.25141 (17)	0.59464 (11)	0.0491 (3)	
H21A	1.1419	0.1668	0.6125	0.059*	
H21B	1.0764	0.3292	0.5718	0.059*	
C22	1.05240 (15)	0.22993 (13)	0.78451 (10)	0.0366 (3)	
C23	1.18780 (16)	0.08993 (14)	0.83232 (10)	0.0392 (3)	
C24	1.19077 (18)	−0.04125 (15)	0.87141 (11)	0.0470 (3)	
H24	1.0985	−0.0538	0.8694	0.056*	
C25	1.3337 (2)	−0.15412 (16)	0.91379 (13)	0.0548 (4)	
H25	1.3372	−0.2432	0.9416	0.066*	
C26	1.4710 (2)	−0.13609 (17)	0.91531 (14)	0.0615 (4)	
H26	1.5660	−0.2133	0.9431	0.074*	
C27	1.46969 (18)	−0.00471 (17)	0.87613 (14)	0.0586 (4)	
H27	1.5621	0.0077	0.8773	0.070*	
C28	1.32679 (16)	0.10650 (15)	0.83550 (11)	0.0438 (3)	
C29	1.13735 (15)	0.32878 (14)	0.76355 (10)	0.0389 (3)	
N1	0.64091 (14)	0.31789 (13)	0.79590 (9)	0.0446 (3)	
N2	0.96439 (13)	0.24825 (11)	0.68832 (8)	0.0386 (3)	
N3	1.29296 (14)	0.24880 (12)	0.79328 (10)	0.0497 (3)	
H3A	1.3632	0.2813	0.7871	0.060*	
O1	0.22311 (19)	0.98242 (14)	0.49209 (11)	0.0914 (5)	
O2	0.69437 (12)	0.49773 (11)	0.89032 (8)	0.0493 (3)	
O3	0.50728 (14)	0.39968 (14)	0.78486 (14)	0.0836 (4)	
O4	0.67564 (14)	0.19579 (13)	0.81793 (11)	0.0701 (4)	
O5	1.07592 (12)	0.45448 (10)	0.72573 (8)	0.0485 (3)	

### Atomic displacement parameters (Å^2^)

**Table:**

	*U*^11^	*U*^22^	*U*^33^	*U*^12^	*U*^13^	*U*^23^
C1	0.128 (2)	0.0676 (13)	0.0613 (12)	−0.0123 (13)	−0.0229 (13)	−0.0090 (10)
C2	0.0710 (11)	0.0486 (9)	0.0556 (10)	−0.0075 (8)	−0.0074 (8)	−0.0206 (7)
C3	0.0848 (13)	0.0505 (9)	0.0519 (9)	−0.0195 (9)	0.0094 (9)	−0.0099 (7)
C4	0.0602 (10)	0.0478 (8)	0.0600 (10)	−0.0199 (7)	0.0149 (8)	−0.0135 (7)
C5	0.0483 (8)	0.0446 (7)	0.0462 (8)	−0.0193 (6)	0.0045 (6)	−0.0199 (6)
C6	0.0531 (9)	0.0602 (9)	0.0457 (8)	−0.0154 (7)	0.0068 (7)	−0.0241 (7)
C7	0.0541 (10)	0.0673 (11)	0.0596 (10)	−0.0054 (8)	0.0015 (8)	−0.0304 (8)
C8	0.0400 (7)	0.0477 (7)	0.0402 (7)	−0.0220 (6)	0.0063 (6)	−0.0187 (6)
C9	0.0339 (6)	0.0453 (7)	0.0378 (7)	−0.0239 (6)	0.0051 (5)	−0.0151 (5)
C10	0.0362 (7)	0.0427 (7)	0.0359 (6)	−0.0236 (6)	0.0053 (5)	−0.0125 (5)
C11	0.0420 (7)	0.0513 (8)	0.0331 (6)	−0.0291 (6)	0.0071 (5)	−0.0134 (6)
C12	0.0474 (8)	0.0644 (9)	0.0392 (7)	−0.0252 (7)	0.0042 (6)	−0.0161 (7)
C13	0.0591 (10)	0.0822 (12)	0.0394 (8)	−0.0333 (9)	−0.0015 (7)	−0.0187 (8)
C14	0.0792 (12)	0.0772 (11)	0.0373 (8)	−0.0457 (10)	0.0069 (8)	−0.0236 (7)
C15	0.0669 (10)	0.0598 (9)	0.0446 (8)	−0.0330 (8)	0.0120 (7)	−0.0241 (7)
C16	0.0484 (8)	0.0513 (8)	0.0378 (7)	−0.0288 (7)	0.0068 (6)	−0.0158 (6)
C17	0.0380 (7)	0.0444 (7)	0.0362 (6)	−0.0213 (6)	0.0051 (5)	−0.0146 (5)
C18	0.0468 (8)	0.0604 (9)	0.0435 (8)	−0.0216 (7)	−0.0022 (6)	−0.0209 (7)
C19	0.0664 (11)	0.0727 (11)	0.0398 (8)	−0.0267 (9)	0.0001 (7)	−0.0228 (7)
C20	0.0666 (11)	0.0744 (11)	0.0442 (8)	−0.0288 (9)	0.0105 (7)	−0.0301 (8)
C21	0.0486 (8)	0.0600 (9)	0.0450 (8)	−0.0253 (7)	0.0138 (6)	−0.0243 (7)
C22	0.0360 (7)	0.0426 (7)	0.0376 (6)	−0.0234 (6)	0.0056 (5)	−0.0144 (5)
C23	0.0392 (7)	0.0450 (7)	0.0379 (7)	−0.0217 (6)	0.0059 (5)	−0.0160 (5)
C24	0.0510 (8)	0.0494 (8)	0.0487 (8)	−0.0293 (7)	0.0091 (6)	−0.0182 (6)
C25	0.0640 (10)	0.0435 (8)	0.0549 (9)	−0.0243 (7)	0.0090 (7)	−0.0145 (7)
C26	0.0498 (9)	0.0505 (9)	0.0681 (11)	−0.0124 (7)	0.0006 (8)	−0.0138 (8)
C27	0.0394 (8)	0.0575 (9)	0.0737 (11)	−0.0205 (7)	0.0006 (7)	−0.0184 (8)
C28	0.0390 (7)	0.0470 (8)	0.0484 (8)	−0.0220 (6)	0.0055 (6)	−0.0169 (6)
C29	0.0387 (7)	0.0455 (7)	0.0396 (7)	−0.0244 (6)	0.0089 (5)	−0.0164 (6)
N1	0.0391 (6)	0.0572 (7)	0.0465 (6)	−0.0290 (6)	0.0070 (5)	−0.0194 (5)
N2	0.0374 (6)	0.0472 (6)	0.0363 (6)	−0.0217 (5)	0.0050 (5)	−0.0167 (5)
N3	0.0365 (6)	0.0502 (7)	0.0676 (8)	−0.0271 (5)	0.0056 (6)	−0.0181 (6)
O1	0.0928 (11)	0.0680 (8)	0.0653 (8)	0.0075 (7)	−0.0170 (7)	−0.0163 (7)
O2	0.0470 (6)	0.0582 (6)	0.0428 (5)	−0.0181 (5)	0.0061 (4)	−0.0235 (5)
O3	0.0361 (6)	0.0715 (8)	0.1459 (13)	−0.0282 (6)	0.0131 (7)	−0.0380 (8)
O4	0.0591 (7)	0.0610 (7)	0.1013 (10)	−0.0415 (6)	0.0089 (7)	−0.0243 (7)
O5	0.0477 (6)	0.0437 (6)	0.0584 (6)	−0.0260 (5)	0.0113 (5)	−0.0166 (5)

### Geometric parameters (Å, º)

**Table:**

C1—O1	1.418 (3)	C16—O2	1.3797 (17)
C1—H1A	0.9600	C17—N2	1.4584 (17)
C1—H1B	0.9600	C17—C18	1.5187 (18)
C1—H1C	0.9600	C17—H17	0.9800
C2—O1	1.365 (2)	C18—C19	1.522 (2)
C2—C3	1.373 (3)	C18—H18A	0.9700
C2—C7	1.377 (3)	C18—H18B	0.9700
C3—C4	1.388 (2)	C19—C20	1.513 (2)
C3—H3	0.9300	C19—H19A	0.9700
C4—C5	1.384 (2)	C19—H19B	0.9700
C4—H4	0.9300	C20—C21	1.523 (2)
C5—C6	1.383 (2)	C20—H20A	0.9700
C5—C8	1.5101 (19)	C20—H20B	0.9700
C6—C7	1.377 (2)	C21—N2	1.4634 (18)
C6—H6	0.9300	C21—H21A	0.9700
C7—H7	0.9300	C21—H21B	0.9700
C8—O2	1.4321 (16)	C22—N2	1.4681 (16)
C8—C9	1.5374 (19)	C22—C23	1.5041 (19)
C8—H8	0.9800	C22—C29	1.5590 (17)
C9—N1	1.5309 (16)	C23—C24	1.3774 (19)
C9—C17	1.5404 (18)	C23—C28	1.3879 (19)
C9—C10	1.5452 (17)	C24—C25	1.385 (2)
C10—C11	1.5146 (18)	C24—H24	0.9300
C10—C22	1.5516 (18)	C25—C26	1.378 (2)
C10—H10	0.9800	C25—H25	0.9300
C11—C16	1.384 (2)	C26—C27	1.386 (2)
C11—C12	1.3929 (19)	C26—H26	0.9300
C12—C13	1.377 (2)	C27—C28	1.375 (2)
C12—H12	0.9300	C27—H27	0.9300
C13—C14	1.379 (3)	C28—N3	1.4060 (18)
C13—H13	0.9300	C29—O5	1.2126 (16)
C14—C15	1.376 (2)	C29—N3	1.3522 (18)
C14—H14	0.9300	N1—O3	1.1997 (17)
C15—C16	1.391 (2)	N1—O4	1.2118 (16)
C15—H15	0.9300	N3—H3A	0.8600
			
O1—C1—H1A	109.5	C18—C17—H17	107.3
O1—C1—H1B	109.5	C9—C17—H17	107.3
H1A—C1—H1B	109.5	C17—C18—C19	107.80 (12)
O1—C1—H1C	109.5	C17—C18—H18A	110.1
H1A—C1—H1C	109.5	C19—C18—H18A	110.1
H1B—C1—H1C	109.5	C17—C18—H18B	110.1
O1—C2—C3	124.88 (18)	C19—C18—H18B	110.1
O1—C2—C7	115.56 (17)	H18A—C18—H18B	108.5
C3—C2—C7	119.55 (16)	C20—C19—C18	111.05 (13)
C2—C3—C4	119.46 (17)	C20—C19—H19A	109.4
C2—C3—H3	120.3	C18—C19—H19A	109.4
C4—C3—H3	120.3	C20—C19—H19B	109.4
C5—C4—C3	121.68 (16)	C18—C19—H19B	109.4
C5—C4—H4	119.2	H19A—C19—H19B	108.0
C3—C4—H4	119.2	C19—C20—C21	112.21 (13)
C6—C5—C4	117.54 (14)	C19—C20—H20A	109.2
C6—C5—C8	122.97 (13)	C21—C20—H20A	109.2
C4—C5—C8	119.43 (14)	C19—C20—H20B	109.2
C7—C6—C5	121.09 (16)	C21—C20—H20B	109.2
C7—C6—H6	119.5	H20A—C20—H20B	107.9
C5—C6—H6	119.5	N2—C21—C20	109.45 (12)
C2—C7—C6	120.55 (17)	N2—C21—H21A	109.8
C2—C7—H7	119.7	C20—C21—H21A	109.8
C6—C7—H7	119.7	N2—C21—H21B	109.8
O2—C8—C5	109.55 (11)	C20—C21—H21B	109.8
O2—C8—C9	109.49 (11)	H21A—C21—H21B	108.2
C5—C8—C9	119.53 (11)	N2—C22—C23	112.92 (11)
O2—C8—H8	105.8	N2—C22—C10	100.87 (10)
C5—C8—H8	105.8	C23—C22—C10	115.71 (10)
C9—C8—H8	105.8	N2—C22—C29	113.85 (10)
N1—C9—C8	112.02 (11)	C23—C22—C29	101.58 (10)
N1—C9—C17	108.63 (10)	C10—C22—C29	112.49 (10)
C8—C9—C17	112.21 (11)	C24—C23—C28	119.79 (13)
N1—C9—C10	108.43 (10)	C24—C23—C22	130.71 (12)
C8—C9—C10	110.21 (10)	C28—C23—C22	109.49 (11)
C17—C9—C10	105.04 (10)	C23—C24—C25	118.73 (14)
C11—C10—C9	113.46 (11)	C23—C24—H24	120.6
C11—C10—C22	118.45 (10)	C25—C24—H24	120.6
C9—C10—C22	104.58 (10)	C26—C25—C24	120.80 (14)
C11—C10—H10	106.5	C26—C25—H25	119.6
C9—C10—H10	106.5	C24—C25—H25	119.6
C22—C10—H10	106.5	C25—C26—C27	121.06 (15)
C16—C11—C12	117.84 (13)	C25—C26—H26	119.5
C16—C11—C10	120.54 (12)	C27—C26—H26	119.5
C12—C11—C10	121.28 (13)	C28—C27—C26	117.54 (15)
C13—C12—C11	121.10 (15)	C28—C27—H27	121.2
C13—C12—H12	119.5	C26—C27—H27	121.2
C11—C12—H12	119.5	C27—C28—C23	122.06 (13)
C12—C13—C14	120.11 (15)	C27—C28—N3	128.86 (13)
C12—C13—H13	119.9	C23—C28—N3	109.08 (12)
C14—C13—H13	119.9	O5—C29—N3	126.03 (12)
C15—C14—C13	120.08 (14)	O5—C29—C22	126.45 (12)
C15—C14—H14	120.0	N3—C29—C22	107.51 (11)
C13—C14—H14	120.0	O3—N1—O4	122.10 (12)
C14—C15—C16	119.42 (15)	O3—N1—C9	119.37 (12)
C14—C15—H15	120.3	O4—N1—C9	118.39 (12)
C16—C15—H15	120.3	C17—N2—C21	111.30 (11)
O2—C16—C11	121.60 (12)	C17—N2—C22	107.05 (10)
O2—C16—C15	117.02 (13)	C21—N2—C22	116.93 (10)
C11—C16—C15	121.37 (14)	C29—N3—C28	112.30 (11)
N2—C17—C18	110.64 (11)	C29—N3—H3A	123.8
N2—C17—C9	104.11 (10)	C28—N3—H3A	123.8
C18—C17—C9	119.60 (11)	C2—O1—C1	118.13 (17)
N2—C17—H17	107.3	C16—O2—C8	112.06 (10)
			
O1—C2—C3—C4	179.39 (17)	C9—C10—C22—C23	−154.69 (10)
C7—C2—C3—C4	0.6 (3)	C11—C10—C22—C29	−38.33 (15)
C2—C3—C4—C5	2.6 (3)	C9—C10—C22—C29	89.17 (12)
C3—C4—C5—C6	−3.7 (3)	N2—C22—C23—C24	−59.95 (18)
C3—C4—C5—C8	178.97 (15)	C10—C22—C23—C24	55.57 (19)
C4—C5—C6—C7	1.7 (2)	C29—C22—C23—C24	177.72 (14)
C8—C5—C6—C7	178.92 (15)	N2—C22—C23—C28	121.06 (12)
O1—C2—C7—C6	178.54 (17)	C10—C22—C23—C28	−123.42 (12)
C3—C2—C7—C6	−2.5 (3)	C29—C22—C23—C28	−1.27 (14)
C5—C6—C7—C2	1.4 (3)	C28—C23—C24—C25	0.4 (2)
C6—C5—C8—O2	−26.75 (18)	C22—C23—C24—C25	−178.55 (13)
C4—C5—C8—O2	150.40 (14)	C23—C24—C25—C26	−1.1 (2)
C6—C5—C8—C9	100.70 (17)	C24—C25—C26—C27	1.0 (3)
C4—C5—C8—C9	−82.15 (18)	C25—C26—C27—C28	−0.2 (3)
O2—C8—C9—N1	63.21 (13)	C26—C27—C28—C23	−0.5 (2)
C5—C8—C9—N1	−64.26 (16)	C26—C27—C28—N3	178.64 (15)
O2—C8—C9—C17	−174.28 (10)	C24—C23—C28—C27	0.5 (2)
C5—C8—C9—C17	58.25 (16)	C22—C23—C28—C27	179.58 (14)
O2—C8—C9—C10	−57.60 (13)	C24—C23—C28—N3	−178.85 (12)
C5—C8—C9—C10	174.92 (11)	C22—C23—C28—N3	0.27 (16)
N1—C9—C10—C11	−102.41 (12)	N2—C22—C29—O5	58.98 (18)
C8—C9—C10—C11	20.53 (14)	C23—C22—C29—O5	−179.33 (13)
C17—C9—C10—C11	141.59 (11)	C10—C22—C29—O5	−54.99 (17)
N1—C9—C10—C22	127.09 (10)	N2—C22—C29—N3	−119.81 (12)
C8—C9—C10—C22	−109.97 (11)	C23—C22—C29—N3	1.87 (13)
C17—C9—C10—C22	11.09 (12)	C10—C22—C29—N3	126.22 (12)
C9—C10—C11—C16	11.56 (17)	C8—C9—N1—O3	22.46 (18)
C22—C10—C11—C16	134.74 (13)	C17—C9—N1—O3	−102.05 (16)
C9—C10—C11—C12	−175.29 (12)	C10—C9—N1—O3	144.30 (14)
C22—C10—C11—C12	−52.11 (17)	C8—C9—N1—O4	−161.64 (12)
C16—C11—C12—C13	0.7 (2)	C17—C9—N1—O4	73.85 (15)
C10—C11—C12—C13	−172.64 (14)	C10—C9—N1—O4	−39.80 (16)
C11—C12—C13—C14	1.8 (3)	C18—C17—N2—C21	63.88 (14)
C12—C13—C14—C15	−2.4 (3)	C9—C17—N2—C21	−166.40 (10)
C13—C14—C15—C16	0.5 (2)	C18—C17—N2—C22	−167.21 (11)
C12—C11—C16—O2	178.36 (12)	C9—C17—N2—C22	−37.49 (12)
C10—C11—C16—O2	−8.27 (19)	C20—C21—N2—C17	−58.79 (15)
C12—C11—C16—C15	−2.6 (2)	C20—C21—N2—C22	177.76 (12)
C10—C11—C16—C15	170.76 (12)	C23—C22—N2—C17	168.07 (10)
C14—C15—C16—O2	−178.89 (13)	C10—C22—N2—C17	43.95 (12)
C14—C15—C16—C11	2.0 (2)	C29—C22—N2—C17	−76.77 (13)
N1—C9—C17—N2	−101.06 (11)	C23—C22—N2—C21	−66.33 (15)
C8—C9—C17—N2	134.53 (11)	C10—C22—N2—C21	169.55 (11)
C10—C9—C17—N2	14.79 (12)	C29—C22—N2—C21	48.83 (16)
N1—C9—C17—C18	23.05 (16)	O5—C29—N3—C28	179.32 (13)
C8—C9—C17—C18	−101.35 (14)	C22—C29—N3—C28	−1.88 (16)
C10—C9—C17—C18	138.91 (12)	C27—C28—N3—C29	−178.18 (15)
N2—C17—C18—C19	−60.27 (15)	C23—C28—N3—C29	1.07 (17)
C9—C17—C18—C19	178.82 (12)	C3—C2—O1—C1	16.0 (3)
C17—C18—C19—C20	54.91 (18)	C7—C2—O1—C1	−165.1 (2)
C18—C19—C20—C21	−53.21 (19)	C11—C16—O2—C8	−30.37 (17)
C19—C20—C21—N2	53.69 (18)	C15—C16—O2—C8	150.56 (13)
C11—C10—C22—N2	−160.01 (11)	C5—C8—O2—C16	−163.66 (11)
C9—C10—C22—N2	−32.51 (12)	C9—C8—O2—C16	63.46 (14)
C11—C10—C22—C23	77.81 (14)		

### Hydrogen-bond geometry (Å, º)

**Table:**

*D*—H···*A*	*D*—H	H···*A*	*D*···*A*	*D*—H···*A*
N3—H3*A*···O3^i^	0.86	2.24	3.085 (2)	167
C14—H14···O5^ii^	0.93	2.54	3.358 (2)	147
C8—H8···O5	0.98	2.40	3.234 (2)	143

Symmetry codes: (i) *x*+1, *y*, *z*; (ii) −*x*+2, −*y*+1, −*z*+2.
